# Design and Development of IoT and Deep Ensemble Learning Based Model for Disease Monitoring and Prediction

**DOI:** 10.3390/diagnostics13111942

**Published:** 2023-06-01

**Authors:** Mareeswari Venkatachala Appa Swamy, Jayalakshmi Periyasamy, Muthamilselvan Thangavel, Surbhi B. Khan, Ahlam Almusharraf, Prasanna Santhanam, Vijayan Ramaraj, Mahmoud Elsisi

**Affiliations:** 1 School of Information Technology and Engineering, Vellore Institute of Technology, Vellore 632014, Tamil Nadu, India; 2 Department of Electrical and Computer Engineering, Lebanese American University, Byblos 13-5053, Lebanon; 3 Department of Data Science, School of Science, Engineering and Environment, University of Sanford, Manchester M5 4WT, UK; 4 Department of Business Administration, College of Business and Administration, Princess Nourah bint Abdulrahman University, P.O. Box 84428, Riyadh 11671, Saudi Arabia; 5 Department of Electrical Engineering, National Kaohsiung University of Science and Technology, Kaohsiung City 807618, Taiwan; 6 Department of Electrical Engineering, Faculty of Engineering (Shoubra), Benha University, 108 Shoubra St., Cairo P.O. Box 11241, Egypt

**Keywords:** IoT, AI, ML, DL, ensemble, GPU, Big Data, contrast agent, diagnostic model

## Abstract

With the rapidly increasing reliance on advances in IoT, we persist towards pushing technology to new heights. From ordering food online to gene editing-based personalized healthcare, disruptive technologies like ML and AI continue to grow beyond our wildest dreams. Early detection and treatment through AI-assisted diagnostic models have outperformed human intelligence. In many cases, these tools can act upon the structured data containing probable symptoms, offer medication schedules based on the appropriate code related to diagnosis conventions, and predict adverse drug effects, if any, in accordance with medications. Utilizing AI and IoT in healthcare has facilitated innumerable benefits like minimizing cost, reducing hospital-obtained infections, decreasing mortality and morbidity etc. DL algorithms have opened up several frontiers by contributing towards healthcare opportunities through their ability to understand and learn from different levels of demonstration and generalization, which is significant in data analysis and interpretation. In contrast to ML which relies more on structured, labeled data and domain expertise to facilitate feature extractions, DL employs human-like cognitive abilities to extract hidden relationships and patterns from uncategorized data. Through the efficient application of DL techniques on the medical dataset, precise prediction, and classification of infectious/rare diseases, avoiding surgeries that can be preventable, minimization of over-dosage of harmful contrast agents for scans and biopsies can be reduced to a greater extent in future. Our study is focused on deploying ensemble deep learning algorithms and IoT devices to design and develop a diagnostic model that can effectively analyze medical Big Data and diagnose diseases by identifying abnormalities in early stages through medical images provided as input. This AI-assisted diagnostic model based on Ensemble Deep learning aims to be a valuable tool for healthcare systems and patients through its ability to diagnose diseases in the initial stages and present valuable insights to facilitate personalized treatment by aggregating the prediction of each base model and generating a final prediction.

## 1. Introduction

Communicable diseases have always been a part of human development from early civilization stages and have been a critical issue threatening human health even till now. Regardless of multiple progressive advances accomplished in medicine and healthcare management, contagious diseases continue to be the primary cause of death, infections, illness, and disability, together with global socio-economic disorders. Accurate and early diagnosis followed up with proper treatment selection can significantly influence the effect of any disease. Several countries worldwide implement a confidential system of management to deal with infectious diseases and their control [1]. With COVID-19 recently added, around 500 infectious diseases have been reported universally. Strategic management approaches are adopted to handle many diseases involving accurate and timely diagnosis of suspected diseases to manage healthcare efficiently. Several elements are involved in an effective disease control system, like accurate diagnosis, prognosis, prediction, treatment, and possible prevention and care. These elements are considered to be vital in facilitating quality clinical service. To realize a wholesome diagnostic system, healthcare providers require complete access to all necessary information to arrive at an accurate diagnosis. Predictions and follow-up treatments can be based only upon the outcome of proper analysis [2,3]. Nevertheless, some numerous challenges and limitations obstruct this from happening in real-world application scenarios:

- Cases where patients might withhold certain significant information that they may consider irrelevant, but it may be critical to arrive at a suitable diagnosis.
- Time and effort are involved in acquiring information.
- Geographical constraints when medical practitioners try to access different information with respect to patients’ health result in an obscured view of their medical documentation [4].
- Depending on updated data whose availability and factors like multiple diseases may be possible, there is an urgency for accurate and timely diagnosis, especially life-threatening diseases, and infections [5].
- Due to the deficiency of suitable diagnostic tools to collect total information about the patient for specific disease types, be it mental health, allergies etc.

With the above-mentioned issues, there is also an aspect worth considering where a diagnosis can be regarded as an evolving factor. The temporal nature of diagnosis is because medical providers need to get more information, as the actual context in which they diagnose, along with probabilities where their predictions might change, must also be considered. For instance, a simple cough could be thought of as a cold or bronchitis, but in recent times, when it worsens over time with other varying symptoms, COVID tests are prescribed [6]. Thus, an AI-based diagnostic system can assist medical providers in finding an effective solution to the issues mentioned above. Furthermore, AI can play an imperative role in assembling all the necessary patient data and can assist medical caregivers in solving patients’ difficulties by categorizing multiple possibilities and aspects that would have been missed otherwise.

DL is a branch of AI which has rapidly been transforming the face of healthcare, presenting the capability to investigate data with higher speed and preciseness that has never been witnessed before. This hierarchical or deep structure-based learning type utilizes layered architecture to study and explore the input data [7]. In DL models, data gets filtered by a cascade of numerous layers, where every succeeding layer uses the output of preceding ones to update its outcomes. DL models can train themselves to be even more accurate as they keep processing more complex data, learning from earlier outcomes to fine-tune and revise their abilities towards making observations concerning existing correlations and associations. DL is based on the biological working of neurons in human brains, where their interconnections and processing of incoming information are imitated in an artificial neural network setup. Analogous to how electrical signals pass through the cells of living organisms that travel through each successive layer of nodes that gets activated as soon as it obtains stimuli from their fellow neurons. In the case of ANNs, that forms the foundation of DL models, where every layer is allocated with a particular segment of input task. Information traverses the multiple layers several times to get treated, refined, and optimized to generate the final desired output. These concealed layers execute the mathematical transformation operations that convert the raw data into resourceful output.

**Figure 1:**
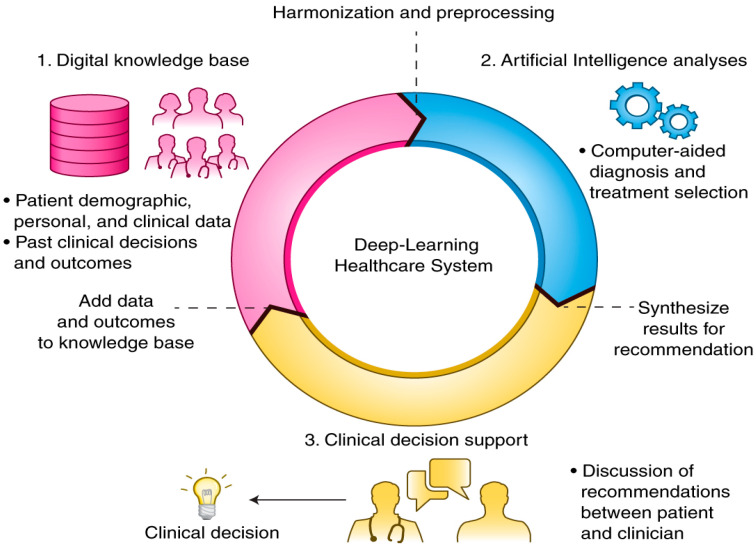
DL in the Healthcare system (Norgeot et al., 2019).

Through the composition of a sufficient number of translations, multiple complex functionalities can be studied and discovered. Upper layers of demonstrations strengthen the aspects of input which are significant for carrying out classification and hold back unrelated disparities. Due to this multi-layered policy, DL models are primarily employed in the medical system (Figure 1) to perform classification tasks like identification of delicate abnormalities present in medical images that would have been missed even by expert practitioners, categorizing the patients with identical attributes to form risk-oriented groups, or highlighting correlations among symptoms and results by analyzing the immense amount of unstructured medical data. In contrast to other ML, DL has the extra leverage of arriving at informed decisions with relatively less or less human interruptions [7,8]. Normal ML [9] procedure necessitates a human trainer/programmer to recognize whether a conclusion is exact. DL, on the other hand, can determine the correctness of its responses on its own owing to its multi-layered framework. Ensemble learning integrates several individual models to attain enhanced generalization and performance. At present, DL architectures are demonstrating promising potential compared to several traditional algorithms. Thus, deep ensemble learning models help leverage the pros of DL [10,11] models and ensemble learning so the ultimate design has better performance and efficiency. The ensemble models are generally grouped into bagging, boosting, stacking etc. The deployment of ensemble ML algorithms to medical imaging results in better accuracy when compared with a single classifier. An enhanced level of accuracy and effectiveness can be achieved by combining different algorithms, enabling a thorough understanding of biological integration, analysis, improved access, and transition in the healthcare domain. This, in turn, will trim down costs, facilitating the earlier discovery of diseases and the exact interpretation of results rather than relying on a single model. Thus, our study focuses on investigating and proposing an ensemble DL and IoT-based medical diagnostic system [12] that lets authorized personnel diagnose disease [13,14]. In addition to diagnosis, our model can incorporate expert facts acquired from multiple sources, which form a knowledge extraction structure that can capture maximum features that are feasible to execute the task of disease characterization. There are two main reasons to use an ensemble-based model in our system over a single model-one aspect is performance, as an ensemble can make better predictions and achieve better performance than any single contributing model. The next one is its robustness, as the ensemble reduces the predictions’ spread or dispersion, thereby improving model performance.

Finally, our contributions can be summarized as follows:

- A novel ensemble DL and IoT-based medical framework [15,16] has been designed and developed to diagnose and predict diseases.
- The deep Ensemble learning technique integrates the predictions of different models to construct a robust model that can reduce variance and avert over-fitting and underfitting phenomena to facilitate accurate prediction of diseases.
- Performance evaluation through extensive experimental results and comparisons are drawn among the suggested and existing models by applying various performance metrics.

The organization of our study is as follows. First, Section 1 gives an overview of the role of DL in healthcare, the challenges faced by traditional approaches, and the benefits of the application of ensemble DL in the medical domain. Section 2 deals with existing research work related to our domain of study. Section 3 provides our design framework and our diagnostic system model in detail. Section 4 presents the evaluation of our system through results analysis and discussion. Finally, Section 5 concludes the paper by highlighting the necessary futures and outlines the scope for future directions.

## 2. Related Works

This section briefly reviews relevant literature works and researches in DL, ensemble learning, and IoT within the medical domain relevant to our proposed model.

Ogundokun et al. (2022) presented a detailed study on various Computational Intelligence approaches that can be applied to diagnosing diseases in the early stages of heart disorders [17,18]. An evaluative analysis between DT and KNN has been provided. The auto-encoder performs feature extraction, minimising the number of attributes needed to portray the heart disease dataset [19]. Another medical IoT-based diagnostic system [20] was proposed by the same author that detects people suffering from the early stages of breast cancer. ANN and CNN with hyper-parameter optimization have been employed to classify tumors to identify if they are malignant or benign, while SVM and MLP were utilized for performing comparisons. The significance of hyper-parameters in influencing the performance of ML models has been emphasized by the authors. Furthermore, feature selection through PSO has been used to enhance classification performance. Still, further meta-heuristic investigations could also be tried to augment the research.

Oyewale et al. [21] listed the limitations encountered by applying traditional techniques in determining the epidemiology of COVID-19. They presented a detailed investigation of the effects of noise filters on the performance of ML algorithms on the dataset. Noise filter algorithms have been utilized to remove noise, and 9 ML techniques have been used to classify and produce accurate results for predicting COVID-19 cases in South Korea. A clear review of several constraints involving classical method-based disease detection has been presented. Yet, it requires further enhancement by exploring more efficient ML and DL models to determine disease epidemiology in real-time using the latest larger datasets.

Choi et al. (2021) designed a prototype using DL models to analyze data from EEG sensors in real time without frequency attributes. This method aimed at predicting stroke disease through DL-based prediction built and trained through data accumulated from sensors in factual applications. The proposed model has been compared with other deep-learning models that are dedicated to prediction based on classification on time series data, and results demonstrated the achievability of alternate methods to gauge brain waves to accurately predict and observe stroke disease in real-life scenarios through lessened cost and efforts in contrast to other existing techniques. However, further in-depth interpretations of various disease predictions using different biological signals like EMG and EEG data need research, and clinically interpretable prediction results through multimodal studies are required to augment the current research study.

Saratxaga et al. (2021) suggested a DL-based image processing method to facilitate MRI-based diagnosis for Alzheimer’s. Balanced accuracy was accomplished for this image-oriented disease diagnosis through automation during the establishment and prediction stages. The results exceeded the conventional techniques that used the OASIS approach. This study reveals that DL-based strategies continue surpassing other tools in designing reliable working models to identify diseases utilizing scanned images from MRI records. It would be even better if further investigations were provided regarding multi-class problems to predict different stages of the disease with high accuracy leading to an emphasis on the effect of reliable diagnosis support tools on extending the life expectancy of patients.

Das et al. (2020) developed a DL-based automated system for disease prediction in rice leaves. This research aims to enhance Crop production through early prediction of crop diseases and to observe appropriate actions to avoid or try not to increase the spread of disease to the areas surrounding the infected plant. This CNN-based system precisely identifies the diseased segment of the leaves by analyzing its images and detaching them through segmentation and classification. On the other hand, training time is more and requires further adaptability of complex functions to fine-tune its performance.

Selvaraj et al. (2019) discussed several research works involved in enhancing the performance of IoT in healthcare systems [22]. Analysis of IoT-based data management techniques in a cloud environment, performance, and systematic study of benefits and constraints in the health industry are evaluated. A review of recent research works which are effective in detecting illnesses and predicting diseases, elderly care, and remote monitoring in healthcare has also been presented in detail. Major drawbacks in the current systems as higher power consumption, restricted availability of lesser resources and security limitations, have also been emphasised. The constraint regarding selecting a particular method to address specific concerns requires further introspection.

Ali et al. (2019) presented a novel healthcare model to predict heart diseases using ensemble DL and feature fusion methods. Feature fusion integrates the features extracted from sensors and EMRs to produce helpful healthcare information [23]. This information gained using this technique eradicates extraneous and unnecessary features and chooses only the significant ones. Dimension reduction results in reduced complexity and improves the efficiency of the system. Moreover, the conditional probability method calculates the exact feature weight for every category and augments the model’s throughput. The ensemble DL model, trained for predicting heart ailments, is evaluated and compared with conventional classifiers. Results show a promising, reliable solution for feature extraction and high-dimensional data set issues. In addition, the risk of bias could have been addressed.

Yasaka et al. (2018) illustrated the fundamental methodological information considering the adaptability of CNNs to perform data collection, implementation, training, and testing in radiology. In addition, constraints encountered regarding this DL technique and how to handle them have been provided meticulously. Moreover, several advanced areas in DL, effects of current scientific studies, potential future opportunities, and directions of medical application of DL techniques have also been provided. Finally, the issue with CNN models regarding orientation and the requirement of larger datasets to train prediction models needs to be addressed.

Vargas et al. (2017) provided a detailed review of recent advancements of DL architectures implemented in several sectors, along with its noteworthy involvement and contribution towards the development of AI. Furthermore, an up-to-date survey on multiple applications and the innovative purposes of DL has been presented together with the strategic arrangement of hierarchy in which layers and non-linear processes are implemented is given. Finally, a comparison with other traditional algorithms and DL’s ever-growing benefits and recognition has been discussed [24].

Ortiz et al. (2016) explored the structure of several DL-based classification methods that can be applied to brain regions to understand and develop a computer-aided diagnosis model for early identification of brain disorders. The acquired images of the brain area are partitioned into multiple spaces, and these patches are employed to guide various deep belief systems. An ensemble of DL networks is created where the ultimate prediction is established through voting. Two DL-based structures and four voting formats are executed and evaluated, thus offering a powerful structural design for classification where discriminative characteristics are estimated in an unsupervised approach.

Bashir et al. (2015) presented a minimum-viable-product in which classification was performed using bagging along with associated weights. The optimized model deals with limitations faced by traditional approaches by employing an ensemble of 7 assorted classifiers. MVP was regression-tested using multiple medical datasets. The analysis concludes that the ensemble framework achieved maximum accuracy in predicting diseases as they showed promising F-Measure and sensitivity compared to architectures that used single classifiers [25].

Most of the existing models suffer from several limitations with respect to speed, performance, accuracy, and reliability. Moreover, the datasets are scarce and suffer from noise and lack personalized medical care. Thus, our objective has been motivated by the aforementioned works to try to offer ensemble methods-based prediction technique that aims to enhance models’ prediction accuracy by integrating the outcomes of multiple models instead of employing a single model. This novel methodology, where combined models are used, can significantly augment the medical diagnostic system’s accuracy and performance.

## 3. Proposed Deep Ensemble Learning Based Disease Prediction Model

This section elaborately discusses the design and architecture of the proposed IoT and deep ensemble learning-based medical diagnostic system for disease prediction. The main motive for utilizing ensemble-based DL in our model is due to the following merits namely:

- Suitable for real-world scenarios where diversity in predictions may often result in inaccurate outcomes affecting the model’s overall performance.
- To choose an optimal model as different algorithms exhibit varied outcomes under varying datasets and offer multiple prediction options.
- Applicable for larger datasets as well as smaller datasets. Instead of training a single classifier with large volumes of data, different classifiers can be used to divide classification tasks. For medical datasets, bootstrapping ensemble strategy can help to create several subsets of a single dataset using a replacement strategy.
- The estimation of confidence through voting further strengthens the prediction outcomes.
- Single classifiers may not be sufficient for situations involving complex problems that require the calculation of polynomial decision boundaries.
- The ensemble learning model is highly recommended for information fusion applications for improving classification performance.
- Drug recommendations in medical applications based on silico analysis of side effects [20]

**Figure 2:**
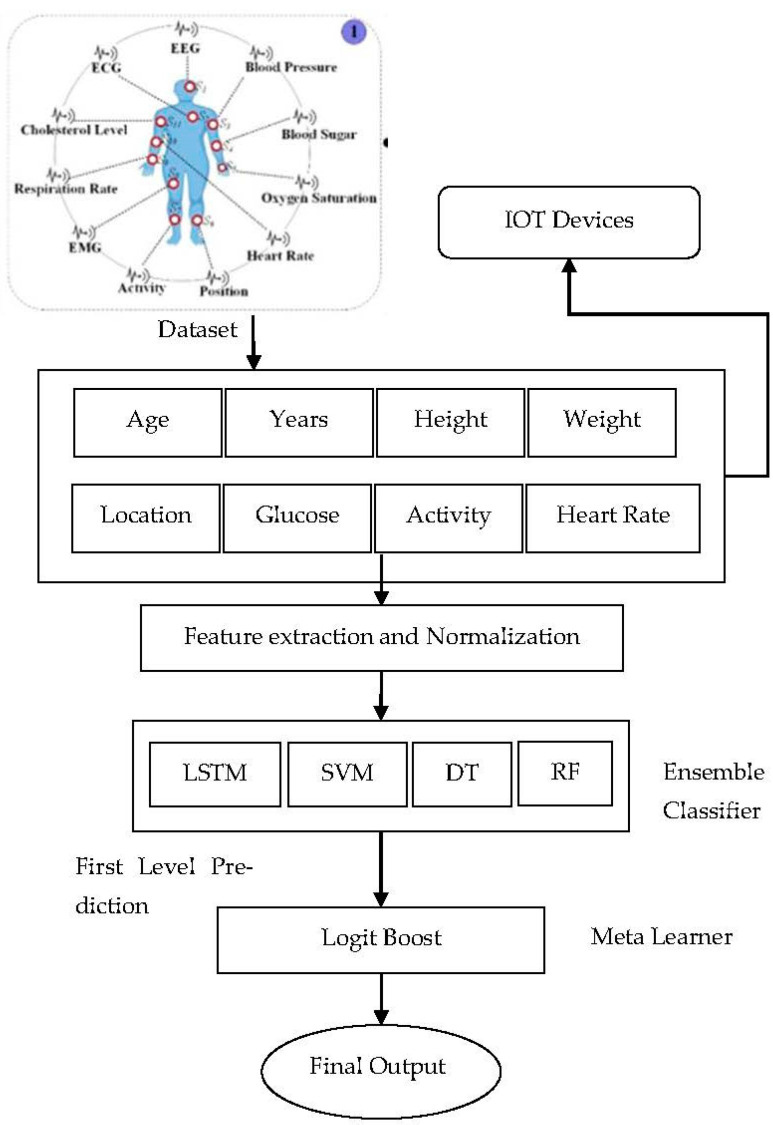
Proposed Deep Ensemble learning architecture for disease prediction.

The architecture (Figure 2) is divided into several layers to facilitate appropriate operations during real-world applications. The ensemble DL model performs the actual prediction in patients and advises on appropriate treatment plans and prognosis. The framework depends on two central data sources-wearable sensors and EMRs. The smart wearable on the user’s body collects interior and exterior physiological information like BP [26,27], glucose level, location, heart, respiration rate, O2 and other vital metrics for monitoring. EMRs act as an essential source containing patients’ medical examination reports, history, diabetes, any other previous illnesses, detailed physiological and radiological examinations, lab reports etc. Once data is collected, our novel scheme transmits data and related information to the concerned gateway devices. Scheme exchanges clinical data using Bluetooth and WI-FI to securely store in a database which can be regarded as Medical Big Data. IoT users like doctors, medical experts, researchers, healthcare providers, and patients can access their results from the database through authorized access [28]. The proposed model aims to predict the probability of a given disease in patients with available health information. Thus, to enhance the overall performance of our proposed model is endowed with essential components, namely:

- IoT-based smart wearable sensor component for data collection.
- Ensemble DL based Diagnostic components to facilitate symptoms-based disease predictions

The information in EMRs usually consists of unnecessary features that may affect prediction accuracy. On the other hand, taking out resourceful information from medical records requires minimizing the noise, choosing only those valuable attributes that achieve precise outcomes, and decreasing the dimensions and complexities of the dataset. Thus, feature selection is crucial for enhancing the clearness of information and reducing the training time of DEL models. Multiple approaches are utilized to perform feature selection in clinical datasets, like

- Rough Sets
- Weighted squares
- Uni-variate Selection
- Sequential forward selection

Our method is based on the concept of IG, which improves accuracy by eliminating noisy characteristics. Out of 10 attributes from our input dataset Ddp, only a few features are considered significant to classify the diseases. The proposed system learns about particular constraints through knowledge based on their significance. Thus, information gain helps in choosing the features necessary to undergo classification. The entropy is employed to measure the uncertainty that calculates the divergence between past and succeeding entropy of input variables, *x* and *y*, as expressed in the following equations:

(1) IG(x∣y)=D(x)−D(x∣y)

*x, y*→Random and discrete variables.

*D(x)*→past entropy function.

(2) $$D(x)=-\sum_{i}\;p\left(x_{i}\right){log}_{2}p{(x}_{i})$$

*p(x_i_)*→Probability of previous occurrence.

Following succeeding entropy-y, conditional entropy for discrete variable x is computed as:

(3) $$\begin{array}{cc} D(x|y) & =-\sum_{i}\;p\left(y_{i}\right)D\left(x|y_{i}\right)\\ & =-\sum_{i}\;p\left(y_{i}\right)\sum_{i}\;\left(p\left(x_{i}|y_{i}\right){log}_{2}p\left(x_{i}|y_{i}\right)\right) \end{array}$$

Thus Information Gain (*IG*) can be calculated using Equations (2) and (3) as:

(4) $$\begin{array}{cc} IG(x|y) & =-\sum_{i}\;p\left(x_{i}\right)X{log}_{2}p{(x}_{i})\\ & =-\left(-\sum_{i}\;p\left(y_{i}\right)\sum_{i}\;\left(p\left(x_{i}|y_{i}\right)X{log}_{2}p\left(x_{i}|y_{i}\right)\right)\right) \end{array}$$

The proposed model calculates approximately the value of every attribute to perform the task of disease prediction through Equation (4). This method removes the less important ones after estimating the *IG* for every feature. Thus, unnecessary features get eliminated one at a time until the fine-tuning of the model happens, resulting in accurate result-based performance. Once data preprocessing is done to extract features from collected data through various processes like filtering, normalization, and feature selection using condition-based probability criteria, pre-processed input is routed to the ensemble DL classifier to obtain the final prediction of diseases.

The Logit Boost algorithm was employed as the learning classifier, which boosts the DL model to attain the highest precision and more benefit in handling noisy data compared with AdaBoost. In addition, it has been developed to minimize bias and variance, thereby enhancing classification accuracy.

The ensemble DL model has five layers:

Input layer-1

Hidden layers 3

Output layer-1

The input layer contains 15 nodes, equivalent to the total features in the input dataset. The hidden layer’s estimation is done in the neural model based on the node count [29]. The number of neurons in a hidden layer which is completely connected is defaulted to 15 to have consistent performance. Key input attributes are allocated to the DNN model by using the input layer. All connected parameters are conceded through 3 hidden layers by increasing associated weights with relevant values from the previous layer. Weighted-sum is then computed, followed by the addition of bias which can be represented as:

(5) $${NetWt}_{j}=\sum_{i}^{n}\;a_{i}\times {Wt}_{i,j}+{bs}_{j}$$

*a_i_*→Input data set.

Wt_i,j_→Associated weights.

*bs_j_*→Associated Bias.

The proposed DL model mostly identifies inactive neurons using activation-function ReLU, which is nothing but a computation of rectified linear-unit. Activation function uses negligible –ve slope 0.01 when the value of ‘a’ is less than ‘0’. This resulted in a leak.

(6) $$f({NetWt}_{j})=\max\left(0,{NetWt}_{j}\right)$$

The transformed data is fed to output nodes to predict the diseases. The output layer has two nodes that represent the outcomes of binary (presence or absence) classification. The training operation of DL is initialized by assigning default preliminary weight to all input datasets. Consequently, the discrepancy between real and expected output is reduced by utilizing a back propagation algorithm. Weights are constantly updated throughout training, expressed as (8).

(7) $$\Delta {wt}_{i,j}=-µ\frac{\partial D}{\partial {wt}_{i,j}}$$

µ Rate at which learning happens.

D→Rate of error.

(8) $$D_{n}=\frac{1}{2}\sum_{P=1}^{n}\;\sum_{O=1}^{n}\;{\left({EO}_{ip}-{AO}_{jq}\right)}^{2}$$

where P,O,TO, and AO denote the count of input samples, outputs, expected result, and actual result, respectively. Errors recorded during training are used to update the weights, and expected results are reassessed consequently. The above steps are reiterated until the negligible error between actual and expected output is attained for the proposed network. In our study, the learning rate is defaulted to 0.02, and the Adam optimizer is used for training the proposed DEL model with noisy and gradient input datasets. The Pseudo code of the proposed scheme is presented in the section below (Algorithm 1):
**Algorithm 1.** Pseudo code for IoT and Ensemble DL-based disease prediction model**Input**: Extracted features
$a=a^{1},a^{2},\ldots a^{n}$ for disease prediction**Output**: Existence or non-existence of diseaseBegin**For each training batch, execute**                  Compute weighted summation, including the value of bias in hidden layers using$\;\sum_{i}^{n}\;a_{i}\times {Wt}_{i,j}+{bs}_{j}$                  Estimate $\Delta {wt}_{i,j}=-µ\frac{\partial D}{\partial {wt}_{i,j}}$ and $D_{n}=\frac{1}{2}\sum_{P=1}^{n}\;\sum_{O=1}^{n}\;{\left({EO}_{ip}-{AO}_{jq}\right)}^{2}$                  Select µ and update
${wt}_{i,j}$
                  Reiterate until the error becomes minimum between
EO and
AO
**End-for**Assign activation fn(ReLU) $f{(NetWt}_{j}$$)=max\left(0,{NetWt}_{j}\right)$ for predicting disease risk. To attain maximum efficiency, employ LogitBoost as the learning classifier

## 4. Results Analysis and Discussion

This section is dedicated to experimental analysis and simulation of the proposed ensemble and IoT-based disease prediction model. Our novel approach is executed on an IntelCorei7-9050H processor with a memory capacity of 8 GB, NVIDIA GTX-105-GPU or equivalent and 64-bit Windows OS. The proposed ensemble model is a feed-forward network using the back-propagation principle and gradient functions like Adam to perform binary classification. ECG, pulse oximeter, and temperature sensors have been used as wearables to collect heart rate, temperature, blood pressure and other medical data. These sensors managed data are captured and saved in the cloud using IoT technology for further analysis. In addition, we utilized features from the generated smart watch-based dataset collected from 25 volunteers, each wearing Amazfit GTS 2 Band watches. These subjects were all of ages ranged from 20 to 70, heights ranged from 5 to 6 ft. and the weight from 50 kg to 95 kgs. The collected data was utilized to train the model and predict the results based on real-time input data (Table 1).

**Figure 3:**
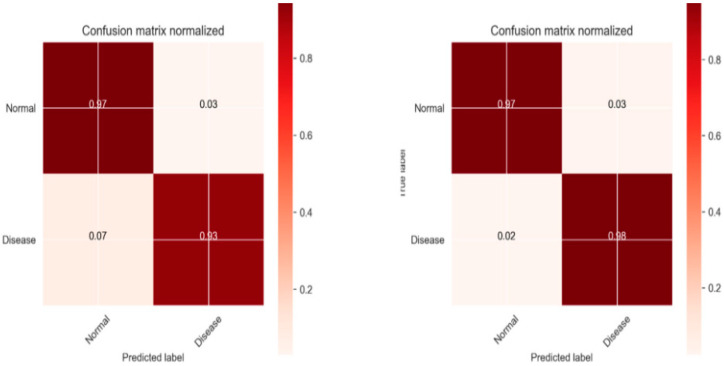
Confusion Matrix.

Ensemble learning combines the prediction of several classification algorithms into distinct representations so that bias along with variation is decreased, thus improving the overall accuracy of the result. Ensemble learning with cross-validation is deployed with SVM, DT and LR classifiers [25]. In the ensemble boosting approach, base-level classifiers are trained through a similar dataset, and a meta-learner is employed to augment the proposed model‘s performance. In the present study, LogitBoost is utilized to improve the accuracy of base-level classification models and generates a final classification result, the binary result (Yes/No) indicating the presence of disease. Model parameters are presented below (Table 2):

The efficiency of any ML model is established by its accuracy. The performance of our proposed model is assessed through metrics like accuracy, specificity, recall, precision, RMSE and MAE. We also performed ablation tests for every ensemble method to validate the effect of each model. This is done by evaluating the performance of ablated ensemble by removing a single model. Table 3 shows the prediction scores for each ablated ensemble and its divergence from the full ensemble. Deep learning methods impacted the ensemble’s performance in a great manner. When the LSTM model was removed, the prediction score dropped the most for the voting and SVM, DT and RF did not affect overall performance much when dropped. Comparative analysis of our novel scheme is provided in Figure 3, a Table 4 from which it can be observed that the results obtained through ensemble modeling have generated significant performance metrics like predictive accuracy being 94.21% in relation to existing methods. In addition, specificity (95.87%), precision (96.48%), and recall (93.65%) confirm the efficacy of our system in comparison to other conventional methods. In addition to that, Table 4 shows the Ablation tests for the ensemble models used in our study.

**Figure 4:**
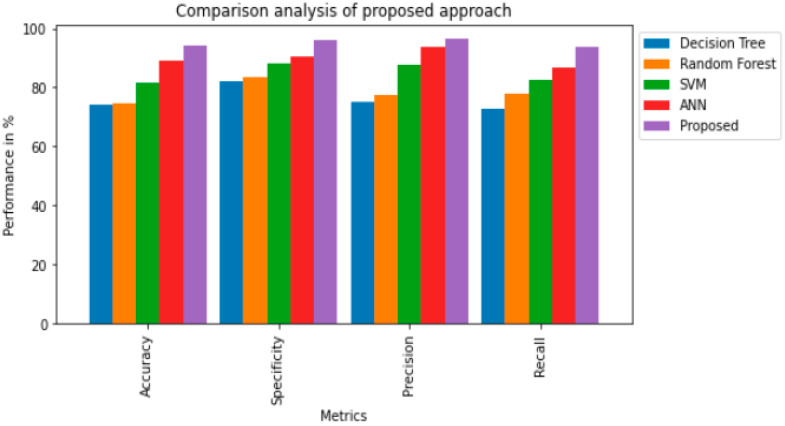
Comparison analysis of the proposed approach.

We have evaluated our suggested method through real-world data, and our experimental results convey that our proposed system produces better performance on medical diagnosis, which has been provided in our current study that also observes its significant efficiency compared to other baseline methods in all factors shown in Figure 4.

Figure 3 shows the confusion matrix before and after tuning hyperparameters. The Proposed algorithm attained better accuracy, 99.3%, compared to the average accuracy of other models. The difference in value was considerable at point *p* = 0.018.

## 5. Conclusions and Future Enhancements

The unbelievable growth of IoT has resulted in a massive boom in Medical Big data, facilitating better healthcare support systems where Patient’s data can be accessed, analyzed, and stored in cloud-based environments. These data are priceless and significant for performing activities like monitoring, diagnosing, and decision-making. Fundamentally, the medical data can be either statistical or pictorial or sequential information with different recovery modes, development, and operation modes. Furthermore, the deployment of Ml and DL in medical care systems is evolving at a larger pace, where several highly effective designs are employed to improve efficiency. Consistency and accuracy are vital aspects in the medical domain, where improving results by preserving stability is always critical [30]. The foremost inspiration for our study has been derived from IoT and ensemble DL techniques. By integrating multiple weak models by means of an ensemble approach, accomplishing accuracy and reliability is possible. We thus propose a deep ensemble learning (DEL) based disease diagnosis and monitoring model which can predict diseases through monitoring from IoT devices. The proposed scheme has been compared with several existing algorithms through various performance evaluation metrics. It has been inferred that our model surpasses them towards achieving a better healthcare support system. In addition, a comparison between our proposed approach and the corresponding individual DL and ML models with similar architecture shows that the proposed approaches outperform the others by fine-tuning the learning rate, activation function, and optimization function to improve the learning process. However, there are limitations like training time and hardware resource consumption as several DL models are ensembled.

On the other hand, this trade-off is trivial as hardware resources are more affordable, and the ultimate requirements of accuracy, consistency and reliability are always the core priorities in healthcare. Another significant feature affecting the prediction has not been addressed, as this is a general limitation of most DL approaches. This approach also reveals that the abovementioned techniques are easily manageable and can be applied to realize optimal efficiency by employing real-time data that can be broadly used in real-world executions. However, as the prime reason to use ensemble models is to deal with noise, bias and variance, there could be situations where the collective experience to improve accuracy may not be feasible, especially in environments where interpretation needs to be generalised and straightforward. In addition, the limitation concerning long inference time and generating overfitting models needs to be considered. Future investigations are dedicated to determining the optimal model parameters to fine-tune the prediction model and check the other possibilities of applying multiple clinical datasets with a larger set of attributes to generate an even more generalized and robust architecture. Furthermore, anomaly detection and data sampling methodologies could also be further considered.

## Figures and Tables

**Table 1:** Dataset Attributes.

S. No	Attributes	Units
1	Age	Years
2	Gender	M/F
3	Height	Inch
4	Weight	Kg
5	Location	-
6	Cholesterol Level	Mg/dL
7	Glucose Level	Mg/dL
8	Activity	h/min/s
9	Resting Data	h/min/s
10	Maximum Heart Rate	Beats/s

**Table 2:** Proposed Model Parameters.

Parameters	Values
Input layer size	Twenty
Output layer size	One
Hidden-layer	Three
Rate of a learning curve	1%
Activation-fn	ReLU
Epochs	100
Optimizers	Adam

**Table 3:** Ablation tests of ensemble methods.

	Voting (%)	
**Method**	**Accuracy**	**Impact**
LSTM	96.15	−0.16
SVM	95.24	−0.05
DT	95.12	−0.02
RF	95.23	−0.06

**Table 4:** Comparison analysis of the proposed approach.

Algorithm	Accuracy (%)	Specificity (%)	Precision (%)	Recall (%)	RMSE (%)	MAE (%)
DT	74.11	82.27	75.02	72.69	42	32
RF	74.66	83.56	77.56	77.89	44	39
SVM	81.58	88.12	87.74	82.56	52	26
ANN	89.29	90.25	93.69	86.54	41	28
Proposed Approach	94.21	95.87	96.48	93.65	33	24

## Data Availability

Not applicable.

## References

[1] Choi Y.-A., Park S.-J., Jun J.-A., Pyo C.-S., Cho K.-H., Lee H.-S., Yu J.-H. (2021). Deep Learning-Based Stroke Disease Prediction System Using Real-Time Bio Signals. Sensors.

[2] Mikolov T., Sutskever I., Chen K., Corrado G.S., Dean J. (2013). Distributed representations of words and phrases and their compositionality. Adv. Neural Inf. Process. Syst..

[3] Saratxaga C.L., Moya I., Picón A., Acosta M., Moreno-Fernandez-De-Leceta A., Garrote E., Bereciartua-Perez A. (2021). MRI deep learning-based solution for Alzheimer’s disease prediction. J. Pers. Med..

[4] Jin K., Yan Y., Wang S., Yang C., Chen M., Liu X., Ye J. (2023). IERM: An Interpretable Deep Learning System to Classify Epiretinal Membrane for Different Optical Coherence Tomography Devices: A Multi-Center Analysis. J. Clin. Med..

[5] Das A., Mallick C., Dutta S. (2020). Deep learning-based automated feature engineering for rice leaf disease prediction. Computational Intelligence in Pattern Recognition.

[6] Pallagani V., Khandelwal V., Chandra B., Udutalapally V., Das D., Mohanty S.P. (2019). DCrop: A deep-learning based framework for accurate prediction of diseases of crops in smart agriculture. Proceedings of the 2019 IEEE International Symposium on Smart Electronic Systems (iSES) (Formerly iNiS).

[7] Kundu N., Rani G., Dhaka V.S., Gupta K., Nayak S.C., Verma S., Ijaz M.F., Woźniak M. (2021). IoT and Interpretable Machine Learning Based Framework for Disease Prediction in Pearl Millet. Sensors.

[8] Ali F., El-Sappagh S., Islam S.R., Kwak D., Ali A., Imran M., Kwak K.-S. (2020). A smart healthcare monitoring system for heart disease prediction based on ensemble deep learning and feature fusion. Inf. Fusion.

[9] Katsaouni N., Tashkandi A., Wiese L., Schulz M.H. (2021). Machine learning based disease prediction from genotype data. Biol. Chem..

[10] Koppu S., Maddikunta PK R., Srivastava G. (2020). Deep learning disease prediction model for use with intelligent robots. Comput. Electr. Eng..

[11] Vargas R., Mosavi A., Ruiz R. (2017). Deep Learning: A Review. https://www.preprints.org/manuscript/201810.0218/v1.

[12] Bashir S., Qamar U., Khan F.H. (2016). IntelliHealth: A medical decision support application using a novel weighted multi-layer classifier ensemble framework. J. Biomed. Informatics.

[13] LaPierre N., Ju C.J.-T., Zhou G., Wang W. (2019). MetaPheno: A critical evaluation of deep learning and machine learning in metagenome-based disease prediction. Methods.

[14] Ortiz A., Munilla J., Górriz J.M., Ramírez J. (2016). Ensembles of Deep Learning Architectures for the Early Diagnosis of the Alzheimer’s Disease. Int. J. Neural Syst..

[15] Kashani M.H., Madanipour M., Nikravan M., Asghari P., Mahdipour E. (2021). A systematic review of IoT in healthcare: Applications, techniques, and trends. J. Netw. Comput. Appl..

[16] Selvaraj S., Sundaravaradhan S. (2020). Challenges and opportunities in IoT healthcare systems: A systematic review. SN Appl. Sci..

[17] Ahmed H., Younis E.M.G., Hendawi A., Ali A.A. (2019). Heart disease identification from patients’ social posts, machine learning solution on Spark. Futur. Gener. Comput. Syst.

[18] Ogundokun R.O., Misra S., Sadiku P.O., Gupta H., Damasevicius R., Maskeliunas R. (2022). Computational Intelligence Approaches for Heart Disease Detection. Recent Innovations in Computing.

[19] Al-Hamadani B. (2016). An Emergency Unit Support System to Diagnose Chronic Heart Failure Embedded with SWRL and Bayesian Network. Int. J. Adv. Comput. Sci. Appl.

[20] Ogundokun R.O., Misra S., Douglas M., Damaševičius R., Maskeliūnas R. (2022). Medical Internet-of-Things Based Breast Cancer Diagnosis Using Hyperparameter-Optimized Neural Networks. Futur. Internet.

[21] Oyewola D.O., Dada E.G., Misra S., Damaševičius R. (2021). Predicting COVID-19 Cases in South Korea with All K-Edited Nearest Neighbors Noise Filter and Machine Learning Techniques. Information.

[22] Al-Makhadmeh Z., Tolba A. (2019). Utilizing IoT wearable medical device for heart disease prediction using higher order Boltzmann model: A classification approach. Measurement.

[23] Ali F., El-Sappagh S., Kwak D. (2019). Fuzzy Ontology and LSTM-Based Text Mining: A Transportation Network Monitoring System for Assisting Travel. Sensors.

[24] Ali F., Islam S.R., Kwak D., Khan P., Ullah N., Yoo S.J., Kwak K.S. (2017). Type-2 fuzzy ontology-aided recommendation systems for IoT-based healthcare. Comput. Commun..

[25] Wang H., Wang K., Xue Q., Peng M., Yin L., Gu X., Wang Y. (2022). Transcranial alternating current stimulation for treating depression: A randomized controlled trial. Brain.

[26] Golino H.F., Amaral L.S.d.B., Duarte S.F.P., Gomes C.M.A., Soares T.D.J., Reis L.A.D., Santos J. (2014). Predicting increased blood pressure using machine learning. J. Obes..

[27] Heo B.M., Ryu K.H. (2018). Prediction of prehypertenison and hypertension based on anthropometry, blood parameters, and spirometry. Int. J. Environ. Res. Public Health.

[28] Karadeniz T., Tokdemir G., Maraş H.H. (2021). Ensemble Methods for Heart Disease Prediction. New Gener. Comput..

[29] Lu S., Yang B., Xiao Y., Liu S., Liu M., Yin L., Zheng W. (2023). Iterative reconstruction of low-dose CT based on differential sparse. Biomed. Signal Process. Control.

[30] Zhang W., Zou H., Luo L., Liu Q., Wu W., Xiao W. (2016). Predicting potential side effects of drugs by recommender methods and ensemble learning. Neurocomputing.

